# Identification of Climatic Factors Affecting the Epidemiology of Human West Nile Virus Infections in Northern Greece

**DOI:** 10.1371/journal.pone.0161510

**Published:** 2016-09-15

**Authors:** Nikolaos I. Stilianakis, Vasileios Syrris, Thomas Petroliagkis, Peeter Pärt, Sandra Gewehr, Stella Kalaitzopoulou, Spiros Mourelatos, Agoritsa Baka, Danai Pervanidou, John Vontas, Christos Hadjichristodoulou

**Affiliations:** 1 Joint Research Centre, European Commission, Ispra (VA), Italy; 2 Department of Biometry and Epidemiology, University of Erlangen-Nuremberg, Erlangen, Germany; 3 Hellenic Centre for Disease Control and Prevention (KEELPNO), Athens, Greece; 4 EcoDevelopment SA, Thessaloniki, Greece; 5 Institute of Molecular Biology and Biotechnology, Foundation for Research and Technology-Hellas, Heraklion, Greece; 6 Department of Hygiene and Epidemiology, Faculty of Medicine, University of Thessaly, Larissa, Greece; Rutgers University, UNITED STATES

## Abstract

Climate can affect the geographic and seasonal patterns of vector-borne disease incidence such as West Nile Virus (WNV) infections. We explore the association between climatic factors and the occurrence of West Nile fever (WNF) or West Nile neuro-invasive disease (WNND) in humans in Northern Greece over the years 2010–2014. Time series over a period of 30 years (1979–2008) of climatic data of air temperature, relative humidity, soil temperature, volumetric soil water content, wind speed, and precipitation representing average climate were obtained utilising the ECMWF’s (European Centre for Medium-Range Weather Forecasts) Re-Analysis (ERA-Interim) system allowing for a homogeneous set of data in time and space. We analysed data of reported human cases of WNF/WNND and *Culex* mosquitoes in Northern Greece. Quantitative assessment resulted in identifying associations between the above climatic variables and reported human cases of WNF/WNND. A substantial fraction of the cases was linked to the upper percentiles of the distribution of air and soil temperature for the period 1979–2008 and the lower percentiles of relative humidity and soil water content. A statistically relevant relationship between the mean weekly value climatic anomalies of wind speed (negative association), relative humidity (negative association) and air temperature (positive association) over 30 years, and reported human cases of WNF/WNND during the period 2010–2014 could be shown. A negative association between the presence of WNV infected *Culex mosquitoes* and wind speed could be identified. The statistically significant associations could also be confirmed for the week the WNF/WNND human cases appear and when a time lag of up to three weeks was considered. Similar statistically significant associations were identified with the weekly anomalies of the maximum and minimum values of the above climatic factors. Utilising the ERA-Interim re-analysis methodology it could be shown that besides air temperature, climatic factors such as soil temperature, relative humidity, soil water content and wind speed may affect the epidemiology of WNV.

## Introduction

The dynamics of vector-borne pathogen transmission are affected by climatic factors. The permanent presence and emergence of vector-borne diseases, the geographic spread of vector species and concerns about the effects of climatic variability and climate change have reinforced the efforts to understand the relation between climate variability and vector-borne infectious diseases [1–3]. Infectious agents and vectors are sensitive to meteorological factors such as temperature, humidity, precipitation, wind and other environmental conditions. Survival and reproduction rates of the mosquito and tick vectors of human pathogens are sensitive to variation in temperature and moisture. The development of infectious agents within vectors (extrinsic incubation) is temperature dependent. Climate variability can lead to changes of seasonal and geographic distribution of vector populations and the diseases [4–6]. These changes can result in altering disease incidence.

West Nile Virus (WNV) is the etiological agent of West Nile Fever (WNF) and West Nile neuro-invasive disease (WNND) in humans. The virus has caused concern since expanding in circulation and becoming endemic in the USA after its introduction in 1999 [7]. WNV originally emerged in Europe and the Mediterranean basin in the 1960s and re-emerged after 1990 when it was responsible for numerous outbreaks in southern and south-eastern Europe through 2010 [8–11]. WNV is a globally distributed virus predominantly transmitted by the *Culex* mosquito species. In Europe primarily *Culex pipiens pipiens* mosquitoes as well as *Culex modestus* and *Culex perxiguus* are vectors of WNV [12]. In Greece *Culex pipiens pipiens* is the dominant vectors of WNV [13]. The transmission cycle that allows the virus to survive in the environment is maintained in an enzootic cycle between mosquitoes and birds. In this cycle mosquitoes acquire WNV after feeding on infected and infectious birds. When the virus reaches the salivary glands of the infected and infectious mosquito it can be transmitted through the bite during the next blood meal to an uninfected bird. Infected birds develop viremia and illness during which the next biting mosquitoes get infected. Infected and infectious mosquitoes bite and infect humans, horses and other mammals, which are dead-end hosts. Infected humans and mammals do not develop sufficient high viremia to pass the virus on to other biting mosquitoes. Although many birds and mammals are susceptible to WNV infection, only some (crows, horses, humans) develop severe and sometimes terminal disease [14].

There is still a lot of uncertainty surrounding the association between meteorological factors and the epidemiology of WNV infections in humans [15]. WNV infections in mosquitoes, birds, veterinary animals and humans are characterised by spatial and temporal variability. Part of the spatial variability is linked to environmental variability including climate [16–18]. Increased temperature in association with decreased precipitation and drought conditions were also associated with heightened WNV infection rates in mosquitoes in regions of the USA [19, 20]. Paz et al. [21] similarly observed a positive association between WNV infections in humans and temperature in southern European and Eurasian countries. It has been observed that the number of WNV infectious mosquitoes and human cases increases after a period of significant temperature deviations towards higher temperatures during summer months [21–23]. The association between precipitation and WNV transmission in humans, mosquitoes and reservoirs species is ambiguous with studies showing both positive and negative correlations [17, 18, 24, 25]. Due to the complexity of the zoonotic transmission cycles no conclusive evidence is expected soon. However, some insights maybe gained if consistent and reliable long term time series of meteorological data with the proper geographical density, validity and precision in association with the corresponding vector and hosts data were available. From the meteorological side, reanalysis data from weather prediction models have become an important source of information [26]. The statistical methodology for the study of spatial and temporal variability of extreme events in climate data has also been developed [27]. Understanding which, how and to what extent environmental conditions affect the epidemiology of WNV infections in humans can result in identifying environmental risk factors, which can inform public health action. Integration of environmental monitoring into to public health surveillance systems will allow for a better assessment of the environmental drivers and the conditions suitable for local pathogen transmission.

We expand the investigation of climatic factors in this context by looking not only at temperature and precipitation, but also at relative humidity, soil temperature, soil water content and wind speed. We analyse data of these climatic factors obtained with the ECMWF’s (European Centre for Medium-Range Weather Forecasts)-Re-Analysis (ERA-Interim) datasets and assess the relationship between reported human cases of WNF/WNND with the above climatic factors including time lag effects related to the emergence of WNF/WNND human cases and the presence of WNV in mosquitoes.

We aim to identify associations of climatic factors with the occurrence of WNF/WNND in humans and WNV infections in *Culex* mosquitoes to uncover climatic conditions that can increase the risk of WNV infections in humans and favour the proliferation of *Culex* mosquitoes. We also investigate the predictive capacity of those factors. We explore these associations and their potential to explain the consecutive and variable WNV outbreaks in Northern Greece in the period 2010–2014.

## Methods

### Epidemiological and entomological data

The study was approved by the ethics scientific committee of the master program for applied public and environmental health of the medical faculty of the University of Thessaly, Greece. Patient information was anonymized and de-identified prior to analysis. In addition, epidemiological data are used at the geographical level of prefectures in Greece (NUTS 3 level according to European Union Nomenclature of Territorial Units of Statistics). Confirmed cases of WNF/WNND in humans reported during the period 2010–2014 in Northern Greece provided by the Hellenic Centre for Disease Control and Prevention is analysed. We consider a total of 441 laboratory confirmed cases independent of clinical type (WN fever, 134 cases, or WN neuro-invasive disease WNND, 307 cases).

**Fig 1:**
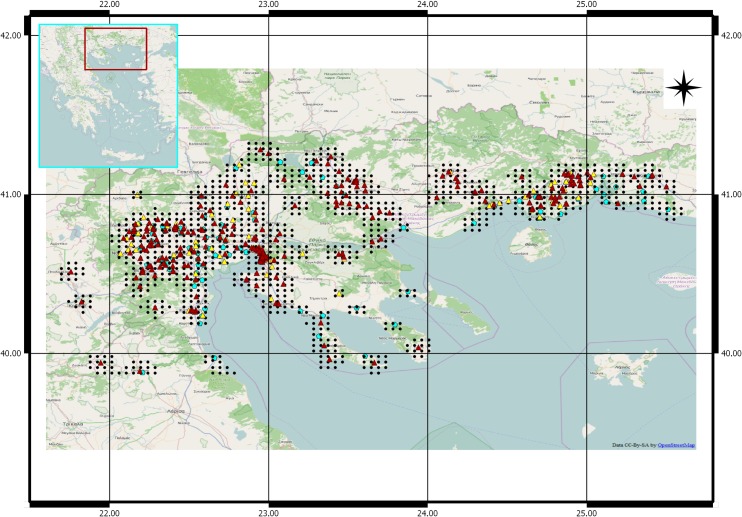
Geographic distribution of the WNF/WNND cases in humans observed during the period 2010–2014 in Northern Greece (triangles) and the grid defined to retrieve the climatic factors based on ERA-Interim (black spots grid). Red triangles represent the neuro-invasive WNND human cases; yellow triangles other milder human cases of WNF; cyan spots mark location of the mosquito traps. (OpenStreetMap software (http://www.openstreetmap.org)

Data obtained from 87 mosquito traps distributed over the same regions for the period 2011–2013 are also analysed with respect to the presence of WNV infected mosquitoes. The majority of the light traps are in the vicinity of stockyards (50–200 m). The rest of the sites are nearby wetlands or drainage canals that serve as mosquito breeding sites. These sites are attractive (stockyards), or productive for *Culex pipiens*, the target species of WNV monitoring. Samples were collected regularly every 15 days, from May to September. Adult mosquitoes were captured inside the trap and were kept alive until they were transferred from the site to the laboratory inside coolers, where they were stored for a maximum of 48 hours in a refrigerator. Identification of a species specific level is based on keys described previously [28, 29]. Groups of 50–100 mosquitoes were deep frozen and were delivered weekly to the microbiology laboratory. Virus screening was performed based on the methodology described previously [30, 31]. The location of the traps can be seen in Fig 1.

### Geographical determination

**Fig 2:**
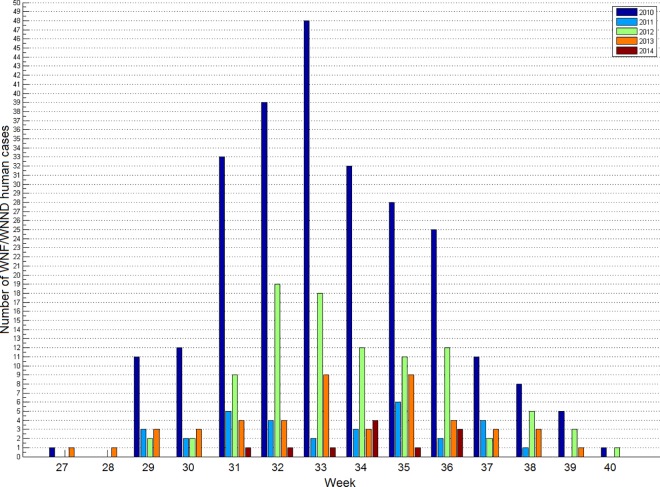
Number of reported WNF/WNND cases in humans for the period 2010–2014 in Northern Greece.

The study area covers an extent of 400 Km x 250 Km. We focus on NUTS3 (Fig 1) areas in Northern Greece since they consistently reported a substantial number (441) of the total number of cases (624) in the country over the 5-year period under consideration (Fig 2). We define a systematic rectangular grid in which the distance between two neighbour points was 0.0345 degrees. We consider only those grids points that surrounded geographically all human cases and mosquito traps. From now on we refer to these points as ‘points of interest’.

### Meteorological data

Time series for air temperature at 2m (T2m, degree Celsius), relative humidity of the air at 2m (RH, %), soil temperature of 1st layer (0 to 7cm, in degrees Celsius), volumetric soil water content of the 1st layer (0 to 7cm; in m^3^ water/m^3^ soil), wind speed (m/s of air flow at 10m), and precipitation (mm/day) were derived from the ECMWF’s (European Centre for Medium-Range Weather Forecasts)-Re-Analysis (ERA-Interim). Reanalysis data from numerical weather prediction (NWP) models have the potential to replace missing weather data in areas of sparse observation points. We employ for our analysis the ECMWF’s (European Centre for Medium-Range Weather Forecasts)-Re-Analysis (ERA-Interim) datasets as they are used in atmospheric sciences where observational data are sparse allowing for a homogeneous set of data in time and space [26]. For the quantification of the agreement between modelled data derived from the ERA-Interim datasets with observational measurements we retrieved data for maximum, mean, and minimum temperature from the National Climatic Data Center/National Oceanic and Atmospheric Administration (NCDC/NOAA, USA) information service [32] and compared the two data sets referring to a period of 30 years (1979–2008). This comparison aims to identify anomalies from the average weekly values. Anomalies are defined as the difference between the weekly average of the climatic factor for the period 1979–2008 and the weekly average of each of the years 2010–2014 divided by the corresponding standard deviation of the period 1979–2008. The approach captures the current changes of the above mentioned climatic factors observed over the last decades.

### The ERA-Interim reanalysis approach

We investigate potential associations between climatic factors and reported human cases of WNF/WNND, and WNV in *Culex* mosquitoes. We built up a set of 36-year (1 January 1979 to 31 December 2014) time series for air temperature, soil temperature, soil water content, relative humidity, total precipitation, and wind speed for 1104 points of interest over Northern Greece utilising the ECMWF’s ERA-Interim reanalysis database. The Global ERA-Interim database, which has been produced by the ECMWF, is the latest global atmospheric reanalysis platform with the capability to provide reliable reanalysis data from numerical weather prediction models [26, 33].

An initial coarse grid (75 Km x 75 Km) attributed to the reanalysis approach had to be adapted to a fined grid (5 Km x 5 Km). In order to construct the desired climatological PDFs (probability density functions), which describe the relative frequency of different data values occurring when sampled from the population of interest, over 1104 points of interest we apply an inverse distance weighting (interpolation) technique that estimates the value of each weather variable resorting to the inverse of the distance to each known point ("amount of proximity"). The final value of the weather variable is calculated (after assigning weights) as the weighted average of the corresponding variable at the 4 surrounding ERA-Interim grid points (nodes). The resulted time series of the weather variables cover the period from 1 January 1979 to 31 December 2014 and are referring to four time windows during each day: (00-06-12-18 UTC, Coordinated Universal Time). The total extent of each time window series is equal to 13140 records (skipping the nine 29 February dates). This set up allows the estimation of the mean (daily) temperature by averaging 00, 06, 12 and 18 UTC values, while at the same time 06 UTC records are considered as resembling the minimum and 12 UTC records the maximum temperature of the day.

**Fig 3:**
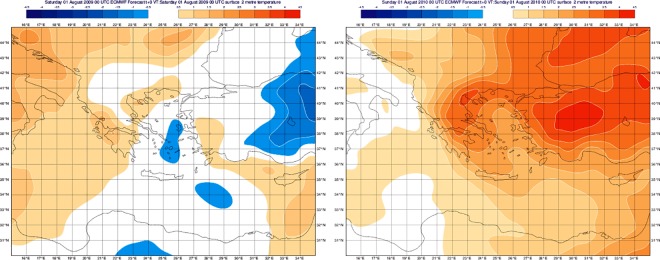
Plots show the air temperature anomalies in August 2009 compared with those of August 2010 the year the first and largest outbreak of WNV infections in Greece.

The first 30 years (1979 to 2008) are used to construct the mean state (“climate”), while the rest of the years are used to identify anomalies. Anomalies denote deviations of climatic factor values over a given period of time when compared to long-term values for the same period of the year. The year 2009 was left out from the averaging process of the climatic anomalies to create some distance to the reference climate. Otherwise, the reference climate would include part of the winter 2009–2010 that was also the winter of 2010, the year where human cases of WNF/WNND occurred. We assume that the winter 2009–2010 may have influenced the mosquito life-cycle and thus the first and largest outbreak of WNV infections took place in Greece. An example of the climatic differences observed in Greece in that period is shown in Fig 3. Mild anomalies of the air temperature in August 2009 were followed by extreme anomalies of temperature in 2010 over the same region and month period.

To explore the reliability of the ERA-interim re-analysis method and its agreement with real measurements we compare long term observations from meteorological stations in Greece on air temperature with the values obtained from the application of the ERA-Interim approach. We exemplify this using the climatic factor for which reliable long term historical data exists, namely air temperature.

We utilise the land-based observations collected by the U.S. NCDC (National Climatic Data Centre: http://www.ncdc.noaa.gov/data-access/land-based-station-data). Land-based observations are collected from instruments sited at locations on every continent. They include temperature, dew point, relative humidity, precipitation, wind speed and direction, visibility, atmospheric pressure, and types of weather occurrences such as hail, fog, and thunder. Data on sub-hourly, hourly, daily, monthly, annual, and multiyear timescales are available.

NCDC data were collected from the cities of Thessaloniki, Larissa, Kavala and Elefsina meteorological stations. We calculate the weekly air temperature average conditions for the periods 1979–2008 and 2009–2014 from the NCDC database and after standardisation we compare them with the corresponding values derived from the ERA-Interim approach. We quantify the agreement between those two measurement methods by using the 95% limits of agreement method [34].

### Statistical analysis

We analysed the association between reported human cases of WVF/WNND and climatic factors and investigated their predictive capacity. Meteorological conditions were expressed in terms of the distribution of the mean data values of the climatic factors under consideration during a 30-year reference period; namely air temperature, soil temperature, soil water content, relative humidity, precipitation, and wind speed. A 30-year period is meteorologically defined as climate ‘normal’ and used to compare current climatological trends to that of the past (World Meteorological Organization, definition). We construct data anomalies and study the changes with respect to that 30-year period.

The values of the climatic factors within the week of the WNF/WNND human cases as well as 1 to 3 weeks prior to their occurrence are used to identify associations. We refine this association by looking at the corresponding 5th, 10th and 90th, 95th percentiles for each climatic parameter and determine the number of WNF/WNND human cases falling in this range. The 10th and 90th percentiles are typically used in meteorology when describing extremes of climatic parameters. We add the 5th and 95th percentiles to also comply with common practice in epidemiology.

The presence or absence of reported human cases of WNF/WNND is used as dependent variable in a multiple logistic regression modelling approach with the anomalies related to wind speed, relative humidity, soil water content, and air temperature and precipitation as explanatory variables. The model associated odd ratios (ORs) and the corresponding 95% confidence interval (CI) are determined. The factors precipitation and soil temperature aren’t taken into consideration in the inferential statistics section since we found it to be correlated with relative humidity (Spearman’s rho = 0.70 and variance inflation factor > 3) and air temperature (Spearman’s rho = 0.93 and variance inflation factor > 6) respectively, a plausible effect due to known meteorological processes. All other parameters do not show any collinearities (variance inflation factor < 3). We create a geographic grid with spatial resolution of 0.0345 degrees (5 Km) over this region and map the WNF/WNND human cases to the points of the grid for which we have values for the climatic factors we analysed. Weekly cases are coded as present or absent. Therefore, there are points on the grid which correspond to more than one cases. This results in a reduction of the number of cases considered in the multiple logistic regression approach used to assess the response to the climatic factors to 399. However, we make full use of all cases in our descriptive statistics results.

We also analyse data on presence/absence of WNV infected *Culex* mosquitoes from 87 traps distributed in Northern Greece over the years 2011–2013, a period where WNF/WNND cases in humans were identified in Greece including the regions under consideration. Employing a multiple logistic regression model using the same approach as with the human cases we explore the association between min, mean, and max value of the parameters and the presence of WNV infected *Culex pipiens* mosquitoes in the traps. A likelihood ratio test provided information about the relative effect of the four environmental factors. In an effort to explore the predictive capacity of these parameters we run the model for the week when human cases of WNF/WNND appeared as well as for a time lag period of one, two and three weeks before. The same is done in the case of the week when a trap was identified to have mosquitoes infected with WNV. For the statistical analysis the JMP software was used. For the statistical analysis the JMP software version 11 (from SAS Institute Inc. Cary, NC, USA) was used. A p-value of < 0.05 is considered to be relevant.

## Results

### Agreement between ERA-Interim and NCDC data

**Fig 4:**
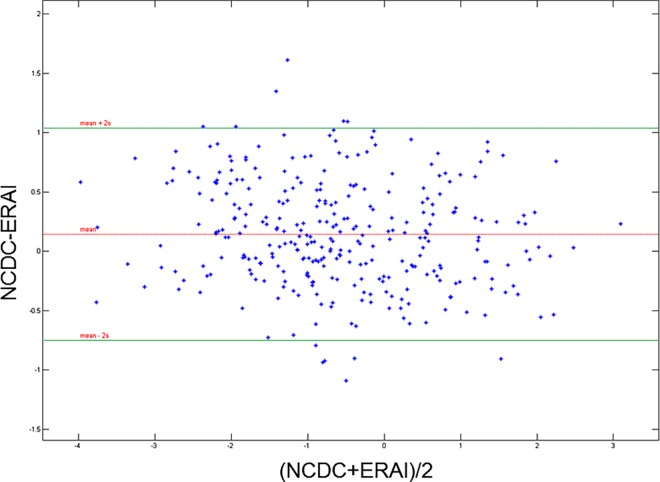
Degree of agreement plot for the air temperature weekly anomalies obtained from the difference of the period 1979–2008 vs. 2010–2014. The difference of NCDC and ERAI against their average and the 2SD range are plotted.

Following the 95% limits of agreement approach [34] we calculated the mean and standard deviation (SD) of the differences between the measurements by the two methods as well as the mean difference ±2SDs. We would then expect 95% of differences between measurements by two methods to lie between these limits. The mean difference of mean temperature weekly anomalies between the two methods (NCDC–ERAI) for the city of Thessaloniki was 0.14 degrees Celsius with a range of ±2SD (-0.75; 1.04) (Fig 4) indicating good degree of agreement. Similar values were obtained for the cities of Larissa, Kavala, and Elefsina when we performed the comparison confirming a good agreement between the two methods of measurement. These results allowed a confident employment of the ERA-Interim method for the assessment of WNF/WNND human cases and the climatic factors under consideration.

### WNF/WNND human cases in Northern Greece

Fig 1 shows the annual distribution of the WNF/WNND cases in Northern Greece for the period 2010–2014. An unexpected large outbreak in 2010 was followed by a less pronounced outbreak in 2011 resurging in 2012 and again two smaller outbreaks in 2013 and 2014, with that of 2014 being the one with the lowest number of cases. All outbreaks ran between week 27 and week 40 of the respective year with some variability in the peaks (week 32–35 depending on the year).

### WNF/WNND human cases and climatic factor percentiles

**Fig 5:**
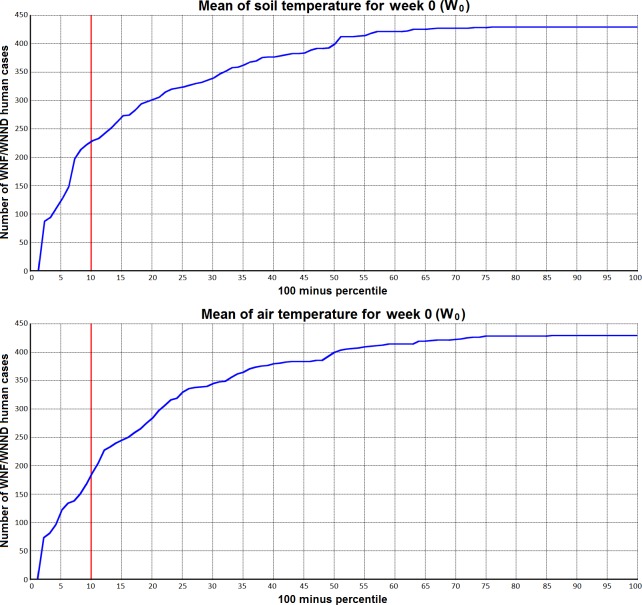
a) For the week (W_0_) the WNF/WNND human case(s) occurred 52% of the cases emerged at soil temperatures above the 90th percentile of the mean value distribution for soil temperature over the period 1979–2008. b) results for air temperature (42%).

**Fig 6:**
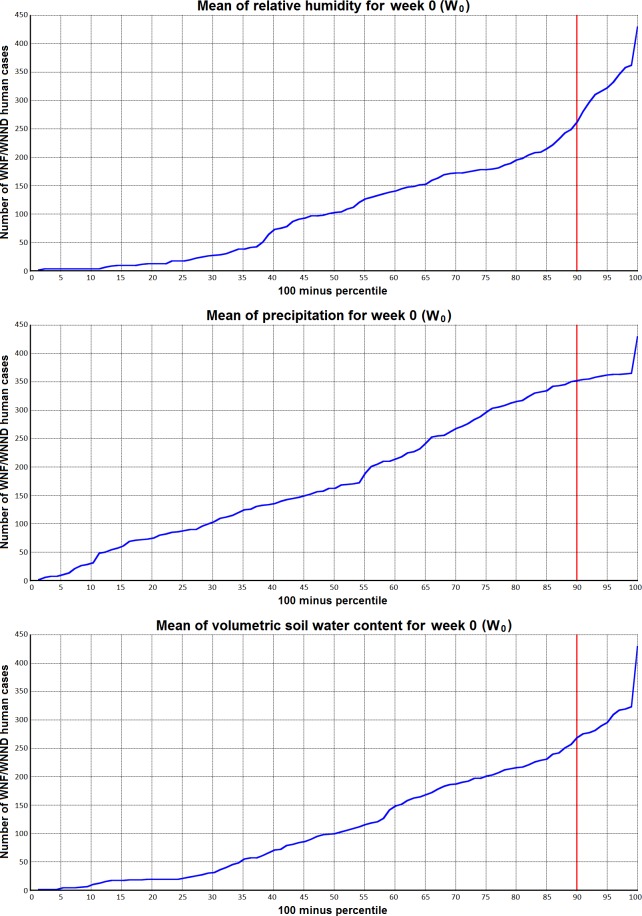
a) For the week (W_0_) the WNF/WNND case(s) occurred 41% of the cases emerged at relative humidity levels above the 10th percentile of the mean value distribution of relative humidity over the period 1979–2008. b) results for precipitation (18%), and c) soil water content (39%).

To evaluate to what extent the extremes of the climatic factors we considered can provide additional valuable information for the risk assessment process we associated the cumulative numbers of WNF/WNND human cases with the distribution of the climatic factors we explore. We place the values of the climatic factors in the distribution of the values of these parameters derived from ERA-Interim over the period 1979–2008 and calculate the cumulative number of WNF/WNND human cases which correspond to the distribution of the climatic factors. The number of cases related to the extreme percentiles, 90th, 95th for the upper and 5th, 10th for the lower percentiles were obtained. This allows an insight into the distribution of the reported WNF/WNND human cases across the percentiles of the climatic variables. Fig 5 and Fig 6 show some typical results. The x-axis on the graph represents the (100-percentile) value of the climatic factor for the week the WNF/WNND human cases occurred. Based on the mean value for soil temperature that was calculated using the ERAI approach for the week (W_0_) when human cases occurred and over all cases which were identified during the period (2010–2014) in Northern Greece, 52% cases (230/441) are predicted to emerge at soil temperatures above the 90^th^ percentile of the soil temperature values for the period 1979–2008 representing the climate of the recent 30 years before the outbreak of the disease in Greece for this variable (Fig 5A). For air temperature the fraction of the cases is 42% (Fig 5B). As the time lag progresses 1–3 weeks backwards the fraction of the cases related to soil or air temperature extremes diminishes slowly.

On the same grounds and looking at the lower extremes relative humidity seems to play an equally important role following the same pattern at the 10th percentile level indicating an inverse relationship between relative humidity and number of WNF/WNND cases in humans (Fig 6A, Table 1).

**Table 1:** Number of WNF/WNND human cases (out of 441) above the 90th and 95th percentile for air temperature and soil temperature and the 5th and 10th percentile for precipitation, relative humidity, soil water content and wind speed of the mean value distributions over the period 1979–2008.

Climatic factors								
Week lag	0	0	1	1	2	2	3	3
								
Percentile	5th	10th	5th	10th	5th	10th	5th	10th
	n (%)	n (%)	n (%)	n (%)	n (%)	n (%)	n (%)	n (%)
Precipitation	70 (16)	80 (18)	55 (12)	63 (14)	59 (13)	70 (16)	48 (11)	55 (12)
Relative humidity	114 (26)	182 (41)	118 (27)	162 (37)	100 (23)	150 (34)	105 (24)	140 (32)
Soil water content	141 (32)	174 (39)	142 (32)	170 (39)	124 (28)	153 (35)	122 (28)	134 (30)
Wind speed	29 (7)	44 (10)	23 (5)	31 (7)	45 (10)	52 (12)	58 (13)	58 (13)
								
Percentile	95th	90th	95th	90th	95th	90th	95th	90th
Air temperature	122 (28)	187 (42)	108 (25)	176 (40)	109 (25)	180 (41)	98 (22)	154 (35)
Soil temperature	128 (29)	230 (52)	119 (27)	217 (49)	111 (25)	197 (45)	113 (26)	172 (39)

Low values of relative humidity were associated with a higher number of human cases (10th percentile, 41%) for the concurrent week of the WNF/WNND human cases emergence as well as all time lags (1–3 weeks). The corresponding value for the 10th percentile of precipitation was 18% (Fig 6B). A more pronounced effect could be attributed to soil water content where even higher numbers of WNF/WNND cases related to the lower percentiles were identified (Fig 6C and Table 1). The number of WNF/WNND cases in association with the 10th percentile ranged between 30% and 39% depending on the time lag and a descending tendency over time. This pattern was observed in all factors we looked at. The extreme values of wind speed were associated with a much lower number of WNF/WNND human cases. The same pattern can be observed with the maximum and minimum values of those climatic factors. Table 1 summarises the number of WNF/WNND cases in humans related to the 90th, 95th and 10th, 5th percentiles respectively of the distribution of the investigated climatic factors.

### Inferential statistics and lag-effects

Using a multiple logistic regression approach we identified associations between the presence of WNF/WNND human cases and the anomalies of the mean weekly values of the climatic factors we explored. For the week the WNF/WNND human cases occurred (lag 0) a clear influence of the anomalies of the mean weekly value of air temperature (p<0.0001), relative humidity (p<0.0001), and wind speed (p<0.0001) on the presence of WNF/WNND human cases could be shown. The same accounted for all time lags (1–3 weeks before the WNF/WNND human cases appeared). This indicates that at least these 3 climatic factors may be of predictive value. The corresponding likelihood ratio test shows that air temperature had the strongest effect followed by relative humidity. Wind speed had a weaker but still statistically significant effect. The relationship is inversely proportional for relative humidity and wind speed meaning a higher probability for the presence of WNF/WNND cases when relative humidity and wind speed are low. Those findings confirm the observations made when we looked at the corresponding associations using the lower extreme percentiles. The odds ratios (OR) for wind speed and relative humidity were 0.76 (95% CI 0.64–0.89) and 0.60 (95% CI 0.53–0.67), respectively, for lag 0. This means that for every unit of increase of wind speed and unit of increase of relative humidity the odds of presence of an infected individual in the concurrent week (W_0_) decreases by 24% and 40% respectively. For time lag of one week (W_-1_) wind speed dependency shows an OR of 0.66 (95% CI 0.56–0.78) for wind speed and 0.67 (95% CI 0.57–0.76) for relative humidity. For lags of 2 and 3 weeks (W_-2_, W_-3_) the OR were 0.74 (95% CI 0.62–0.86) and 0.72 (95% CI 0.61–0.85) for wind speed and 0.72 (95% CI 0.64–0.82) and 0.83 (95% CI 0.74–0.94) for relative humidity respectively.

Air temperature is positively associated with the detection of WNF/WNND cases in humans. Every unit of air temperature (degrees Celsius) increased the odds of presence of an infected individual in the concurrent week (W_0_) by the factor 1.48 (95% CI 1.36–1.62). The corresponding values for W_-1_ are 1.51 (95% CI 1.38–1.64) for W_-1_, 1.37 (95% CI 1.26–1.48) for W_-2_ and 1.25 (95% CI 1.15–1.35) for W_-3_. The results indicate a risk reduction with backwards time lag. Soil water content has also an inverse relationship to the presence of WNF/WNND human cases as well as relative humidity and wind speed. The association was not statistically significant though. Results are summarized in Table 2.

**Table 2:** Odds ratios for the risk of the presence of WNF/WNND human cases in association with anomalies of the mean weekly values for relative humidity, air temperature, soil water content and wind speed for the concurrent week 0 WNF/WNND human cases occurred and for time lags 1–3 weeks.

Climatic factors				
Week lag	0	1	2	3
	OR (95% CI)	OR (95% CI)	OR (95% CI)	OR (95% CI)
Relative humidity	0.60 (0.53–0.67)*	0.67 (0.57–0.76)*	0.72 (0.64–0.82)*	0.83 (0.74–0.94)*
Soil water content	0.96 (0.92–1.01)	0.99 (0.95–1.04)	0.99 (0.94–1.03)	0.97 (0.93–1.02)
Wind speed	0.76 (0.64–0.89)*	0.66 (0.56–0.78)*	0.74 (0.62–0.86)*	0.72 (0.61–0.85)*
**Air temperature**	1.48 (1.36–1.62)*	1.51 (1.38–1.64)*	1.37 (1.26–1.48)*	1.25 (1.15–1.35)*

OR = Odds Ratio

*p<0.0001

Similar statistically relevant associations between the risk of presence of human cases of WNF/WNND infections and anomalies related to the maximum weekly values of the meteorological variables under consideration could be identified for soil water content, OR 0.87 (95% CI 0.83–0.92) for W_0,_ and OR 0.91 (95% CI 0.87–0.96); OR 0.92 (95% CI 0.87–0.97); and OR 0.92 (95% CI 0.88–0.97) for W_-1_, W_-2_, W_-3,_ (p<0.001). For air temperature the OR was 1.33 (95% CI 1.20–1.47); 1.32 (95% CI 1.20–1.46); 1.21 (95% CI 1.10–1.34), (p<0.0001) for W_0_, W_-1_, W_-2_, and 1.10 (95% CI 1.01–1.22), (p<0.05) for W_-3_ implying that anomalies of maximum values of the above factors can also have a considerable predictive value. Based on the likelihood ratio test the effects of these factors are greater for air temperature during the concurrent back to previous weeks (W_0_, W_-1_ W_-2_) but not for W_-3_ where soil water content is dominant. The minimum anomaly values show the same trends for the same parameters as also the maximum anomalies do.

Using a multiple logistic regression analysis, we also explore the relationship between the presence/absence of WNV infected mosquitoes obtained from mosquito traps distributed in the geographic area where the WNF/WNND human cases were identified and the corresponding mean weekly values of the anomalies of the climatic factors. WNV infected mosquitoes were found in 19 of 87 traps. A clear statistical association between wind speed and the presence of WNV infected mosquitoes in traps during the concurrent week (W_0_) with WNF/WNND human cases emerged (p<0.019) was identified. The dependence expressed in the form of the regression coefficient is negative indicating an inversely proportional relationship between traps containing WNV infected mosquitoes and high wind speed. For every unit of wind speed increase the odds for the presence of a trap with WNV infected mosquitoes decreases by 76% (OR 0.24; 95% CI 0.06–0.80). For lags in time of 1–3 weeks no statistically relevant association could be found. The relationship was always inversely proportional for all lags (1–3 weeks). No association could be demonstrated between traps with WNV infected mosquitoes and air temperature, relative humidity or soil water content neither for the concurrent week nor for the other lag-times (1–3 weeks). Wind speed may at least play a role during weeks WNV infected mosquitoes were collected. When looked at the maximum of minimum values of these climatic factors no statistical association with the presence of WNV infected mosquitoes could be shown.

## Discussion

The consecutive outbreaks of WNV transmission in humans observed in southern and south- eastern Europe in recent years beginning in 2010 were associated with climatic drivers, in particular air temperature, supporting the establishment of WNV in new areas through the geographic expansion and abundance of the vector species [15].

Several studies using statistical and mathematical modelling approaches looked at the identification of environmental and other factors of WNV transmission in birds, horses and humans. Paz et al. [22] provided a comprehensive review of environmental drivers of WNV epidemiology and Chevalier et al., [35] did the same for predictive modelling approaches. The vast majority of the studies focusses on air temperature as the most prominent environmental and weather condition followed by precipitation and relative humidity, with the latter showing very weak, if any, associations with WNV infections in mosquitoes, birds or humans. The results of the studies are contradictory indicating that it is difficult to disentangle the individual effects of these factors due to the complexity of the zoonotic transmission cycles. However, collection of reliable and long term meteorological data and modelling techniques may allow us to shed some light on the complex interactions between vectors, amplifiers (such as birds), dead-end hosts and climatic conditions. Looking at more meteorological factors that may be of greater relevance for these interactions is worthwhile in the future.

The aim of our study was to investigate these associations by taking into account more meteorological parameters over long periods of time with complete spatial and temporal density. One of the strengths of our study is the use of the ERA-Interim re-analysis methodology, a robust modelling framework, that allows us to replace missing weather data in areas of sparse observation points and have a homogeneous set of data in time and space. We utilized information on meteorological variables that, due to their unavailability, weren’t used before but may be of relevance for the association between the epidemiology of WNV infections and climate. Our comparison of real observations of air temperature with the values obtained from modelling showed that the use of the latter can be a reasonable approximation. In that way, we demonstrate that climatic data reanalysed from atmospheric modelling when jointly studied with vector-borne diseases data can contribute to the understanding of the underlying associations between climate and the vector-borne pathogen transmission dynamics.

A salient feature and major advantage of our study is the identification of direct associations between climatic factors and WNF/WNND human cases, whereas most of the studies show the association between climatic factors with mosquito density rather than human cases.

The distribution of the WNF/WNND human cases in the period 2010–2014 in Northern Greece shows limited variability in duration and the time of emergence but higher variability in number of cases with the initial year 2010 being most striking. Climatic factors such as air temperature reached significantly higher values in the summer 2010 compared to the corresponding period of 2009. The intermediate years showed anomalies towards higher temperatures with the last year 2014 being very variable in precipitation and temperature, something that might have influenced the developmental dynamics of the mosquitoes and as well as their abundance, distribution and activity.

The comparison of the number of WNF/WNND cases related to the extreme upper percentiles of the distributions of the climatic factors we investigated confirmed that soil and air temperature were associated with a considerable number of cases making these two factors valuable candidates to serve as predictors of outbreaks. Although the association between increased air temperature and elevated WNV transmission in birds, due to the sensitivity of the mosquito to ambient conditions, has been described by other authors [36], we could show this association with reported human cases of WHF/WNND and provide information on the association with soil temperature. Even if the two variables are correlated due to their underlying physical processes the results suggested that soil temperature may be more relevant to the ecology of the disease underlying the sensitivity of the vector to soil temperature. Our results indicate that the highest impact is made by soil temperature followed by air temperature and soil water content. Soil temperature may be directly responsible for the increase in larval development and reduced generation time, whereas air temperature may be more responsible for the extrinsic incubation period of the mosquitoes. Changes in air and soil temperatures might affect WNV hosts such as birds and thus WNV transmission. Observations indicate an earlier migration of birds to their breeding sites as a consequence of early rise of the mean spring temperatures [37].

We also observed an association between the occurrence of WNF/WNND human cases with relative humidity, precipitation and soil water content at their lower percentile bounds. These findings confirmed by the statistically significant associations we found between WNF/WNND human cases and relative humidity and soil water content allow us to infer that water related conditions are inversely associated with the emergence of WNF/WNND cases in humans. Our results indicate that relative humidity, precipitation and soil water content may be additional environmental factors also playing an important role in the transmission dynamics of WNV. With respect to precipitation, although the disease prevalence may be influenced by high precipitation the complicated interaction with the ecology of the vectors doesn’t allow any conclusions [38]. The negative association we find between relative humidity and WHF/WNND cases is in contrast to other studies showing positive associations [39]. Given the correlation between relative humidity and precipitation due to the underlying physical processes and the influence of the above mentioned ecological component of the vectors on the zoonotic transmission cycle of the pathogen, this discrepancy is no surprise. Scarcity of water seems to be a boosting factor for the transmission of WNV and therefore for the occurrence of WNF/WNND cases in humans. Droughts increase the density of birds and mosquitoes around water sources left and thus they may accelerate the transmission of WNV within these populations [40]. Thus our findings confirm previous observations that drought occurrence may increase the risk of WNV outbreaks in humans [41] and point to soil water content as a potential and maybe better predictor compared with relative humidity and precipitation. These results are based on three different water related climatic factors and therefore constitute evidence and are worth investigating further.

In addition, we find that low wind speed is associated with the presence of WNV infected mosquitoes in traps in the concurrent week of the emergence of WNF/WNND human cases. High wind speeds may result in a lower chance for a blood meal and thus in lower WNV infections in humans.

Interestingly, our results imply that the anomalies of the maximum and minimum values may provide additional confidence to predictions and they also indicate the potential of other parameters. The long term predictive capacity of soil water content, for instance, when one looks at maximum and minimum temperatures is of interest. Similarly, the role of wind speed needs further investigation. Moreover, one may expand our methodology of looking at the climatic factor percentiles by building clusters of weeks at which WNF/WNND human cases occurred and link them to the percentiles of a combination of climatic factors. Information about the density of *Culex* mosquitoes in Northern Greece in association with WNV before the 2010–2014 outbreaks in Greece was not available. The number of human WNF/WNND cases is relatively small in relation to the geographic region we investigated. We couldn’t identify any spatial auto-correlation effects with our approach. However, we cannot exclude their existence entirely. Although our approach is a statistical one and doesn’t allow for conclusions on causality the results indicate new directions on exploring the association of WNV infections in humans and climate.

Mathematical modelling approaches in combination with statistical methods and coupling of the transmission processes to the environmental drivers such as meteorological factors may give insights into the underlying dynamical processes. It is likely that a combination of several meteorological parameters can prove very valuable in this context. Better estimates of these parameters and modern mathematical modelling techniques will contribute to a better understanding of the links between environmental factors and the emergence of vector-borne infectious diseases.

## References

[1] Kilpatrick AM, Randolph SE. Drivers, dynamics, and control of emerging vector-borne zoonotic diseases. Lancet. 2012; 380:1946–1955. 10.1016/S0140-6736(12)61151-9 23200503 PMC3739480

[2] Smith KR, Woodward A, Campbell-Lendrum D, Chadee DD, Honda Y, Liu Q, et al Human health: impacts, adaptation and cobenefits In: Climate Change 2014: Impacts Adaptation and Vulnerability. Part A: Global and Sectoral Aspects. Contribution of Working Group II to the Fifth Assessment Report of the Intergovernmental Panel on Climate Change [Filed CB, Barros VR, Dokken DJ, Mach KJ, Mastrandrea MD, Bilir TE, et al (eds.)], Cambridge University Press, Cambridge, United Kingdom and New York, NY, USA, pp. 709–754, 2014.

[3] Semenza JC, Menne B. Climate change and infectious diseases in Europe. Lancet Infect Dis. 2009; 9:365–375. 10.1016/S1473-3099(09)70104-5 19467476

[4] Cardenas R, Sandoval CM, Rodriguez-Morales AJ, Vivas P. Zoonoses and climate variability. Ann NY Acad Sci. 2008; 1149: 326–330. 10.1196/annals.1428.094 19120241

[5] Medlock JM, Leach SA. Effect of climate change on vector-borne disease risk in the UK. Lancet Infect Dis. 2015; 15:721–730. 10.1016/S1473-3099(15)70091-5 25808458

[6] Lafferty KD. The ecology of climate change and infectious diseases. Ecology 2009; 90: 888–900. 19449681 10.1890/08-0079.1

[7] Marfin AA, Gubler DJ. West Nile encephalitis: an emerging disease in the United States. Clin Infect Dis. 2001; 33:1713–1719. 11595987 10.1086/322700

[8] Hayes EB, Komar N, Nasci RS, Montgomery SP, O’Leary DR, Campbell GL. Epidemiology and transmission dynamics of West Nile virus disease. Emerg Infect Dis. 2005; 11:1167–1173. 16102302 10.3201/eid1108.050289a PMC3320478

[9] Mann BR, McMullen AR, Swetnam DM, Salvato V, Reyna M, Guzman H, et al Continued evolution of West Nile Virus, Houston Texas, USA 2002–2012. Emerg Infect Dis. 2013; 19:1418–1426. 10.3201/eid1909.130377 23965756 PMC3810927

[10] Marka A, Diamandidis A, Papa A, Valiakos G, Chaintoutis SC, Doukas D, et al for the MALWEST project. West Nile Virus State of the Art Report of MALWEST Project. Int J Environ Res Public Health. 2013; 10:6534–6610. 10.3390/ijerph10126534 24317379 PMC3881129

[11] Zeller HG, Schuffenecker I. West Nile virus: an overview of its spread in Europe and the Mediterranean basin in contrast to its spread in in the America. Eur J Clin Microbiol Infect Dis. 2004; 23:147–156. 14986160 10.1007/s10096-003-1085-1

[12] Engler O, Savini G, Papa A, Figuerola J, Groschup MH, Kampen H, et al European Surveillance for West Nile Virus in Mosquito Populations. Int J Environ Res Public Health. 2013; 10:4869–4895. 10.3390/ijerph10104869 24157510 PMC3823308

[13] Papa A, Xanthopoulou K, Tsioka A, Kalaitzopoulou S, Mourelatos S. West Nile Virus in mosquitoes in Greece. Parasitol Res 2013; 112;1551–1555. 10.1007/s00436-013-3302-x 23371497

[14] Colpitts TM, Conway MJ, Montogomery RR, Fikrig E. West Nile Virus: Biology, Transmission, and Human Infection. Clin Microbiol Rev 2012; 25:635–648. 10.1128/CMR.00045-12 23034323 PMC3485754

[15] Paz S, Semenza JC. Environmental Drivers of West Nile Fever epidemiology in Europe and Western Asia–A review. Int J Environ Res Public Health. 2013; 10:3543–3562. 10.3390/ijerph10083543 23939389 PMC3774453

[16] Soverow JE, Wellenious GA, Fisman DN, Mittleman MA. Infectious Disease in a warming world: how weather influenced West Nile Virus in the United States (2001–2005). Environ Health Perspect. 2009; 117:1049–1052. 10.1289/ehp.0800487 19654911 PMC2717128

[17] Chung WM, Buseman CM, Joyner SN, Hughes SM, Formby TB, Luby JP, et al The 2012 West Nile encephalitis epidemic in Dallas Texas. JAMA. 2013; 310:297–307. 10.1001/jama.2013.8267 23860988

[18] Wimberly MC, Lamsal A, Giacomo PC, Chuang-TW. Regional variation of climatic influences on West Nile Virus outbreaks in the United States. Am J Trop Med Hyg. 2014; 91:677–684. 10.4269/ajtmh.14-0239 25092814 PMC4183387

[19] Johnson BJ, Sukhdeo MV. Drought-induced amplification of local and regional West Nile virus infection rates in New Jersey. J Med Entomol. 2013; 50:195–204. 23427670 10.1603/me12035

[20] Ruiz M, Chaves L, Hamer G, Sun T, Brown W, Walker E, et al Local impact of temperature and precipitation on West Nile virus infection in Culex species mosquitoes in northeast Illinois, USA. Parasit Vectors. 2010; 3:19 10.1186/1756-3305-3-19 20302617 PMC2856545

[21] Paz S, Malkinson D, Green MS, Tsioni G; Papa A Danis K, et al Permissive summer temperatures of the 2010 European West Nile fever upsurge. PLoS ONE 2013 8(2): e56398 10.1371/journal.pon.0056398 23431374 PMC3576399

[22] Hartley DM, Barker CM, Le Menach A, Niu T, Gaff HD, Reisen WK. Effects of temperature on emergence and seasonality of West Nile Virus in California. Am J Trop Med Hyg. 2012; 86:884–894. 10.4269/ajtmh.2012.11-0342 22556092 PMC3335698

[23] Hahn MB, Monaghan AJ, Hayden MH, Eisen RJ, Delorey MJ, Lindsey NP, et al M. Meteorological condition associated with increased incidence of West Nile Virus disease in the United States. Am J Trop Med Hyg. 2015; 92:1013–1022. 10.4269/ajtmh.14-0737 25802435 PMC4426558

[24] Crowder DW, Dykstra EA, Brauner JM, Duffy A, Reed C, Martin E, et al West Nile Virus prevalence across landscapes is mediated by local effects of agriculture on vector and host communities. PLoS ONE. 2013; 8(1):e55006 10.1371/journal.pone.0055006 23383032 PMC3559328

[25] Deichmeister JM, Telang A. Abundance of West Nile Virus mosquito vectors in relation to climate and landscape variables. J Vector Ecol. 2011; 36: 75–85. 10.1111/j.1948-7134.2011.00143.x 21635644

[26] Dee DP, Uppala SM, Simmons AJ, Berrisford P, Poli P, Kobyashi S, et al The ERA-Interim reanalysis: configuration and performance of data assimilation system. Q J R Meteorol Soc. 2011; 137:553–597.

[27] Coelho CAS, Ferro CAT, Stephenson DB. Methods for exploring spatial and temporal variability of extreme events in climate data. J Clim. 2008; 21: 2072–2092.

[28] Darsie RF Jr, Samanidou-Voyadjoglou A. Keys for the identification of the mosquitoes of Greece. J Am Mosq Control Assoc. 1997; 13: 247–254. 9383766

[29] Becker N, Petric N., Zgomba M, Boase C, Minoo M, Dahl C, et al Mosquitoes and their control, Berlin-Heidelberg, Springer; 2010

[30] Papa A, Papadopoulou E, Gavana E, Kalaitzopoulou S, Mourelatos S. Detection of West Nile virus lineage 2 in Culex mosquitoes, Greece, 2012. Vector Borne Zoonotic Dis. 2013; 13: 682–684 10.1089/vbz.2012.1212 23697769

[31] Papa A, Xanthopoulou K, Gewehr S, Mourelatos S. Detection of West Nile Virus lineage 2 in mosquitoes during a human outbreak in Greece. Clin Microbiol Infect. 2011; 17: 1176–1180. 10.1111/j.1469-0691.2010.03438.x 21781205

[32] National Oceanic and Atmospheric Administration USA (2015): NCDC climate data online. *http://www.ncdc.noaa.gov/cdo-web/*

[33] Berrisford P, Dee D, Fielding K, Fuentes M, Kallberg P, Kobayashi S, et al. The ERA-INTERIM archive. ERA Report Series. No. 1. Technical (ERA) Report. European Centre for Medium-range Weather Forecasting, Shinfield Park, Reading, U.K. 2009

[34] Bland JM, Altman DG. Measuring agreement in method comparison studies. Stat Methods Med Res. 1999; 8: 135–160. 10501650 10.1177/096228029900800204

[35] Chevalier V, Tran A, Durand B. Predictive modelling of West Nile Virus transmission risk in the Mediterranean Basin: how far from landing? Int J Environ Res Public Health. 2014; 11: 67–90. 10.3390/ijerph110100067 PMC3924437 24362544

[36] Reisen WK, Fang Y, Martinez VM. Effects of temperature on the transmission of West Nile virus by Culex trsalis (Diptera:Culicidae). J Med Entomol. 2006; 43: 309–317. 16619616 10.1603/0022-2585(2006)043[0309:EOTOTT]2.0.CO;2

[37] Sparks T, Bairlein F, Bojarinova J, Hüppop O, Lehikoinen E, Rainio K, et al Examining the total arrival distribution of migratory birds. Glob Change Biol. 2005; 11:22–30.

[38] Takeda T, Whitehouse CA, Brewer M, Gettman AD, Mather TN. Arbovirus surveillance in Rhode Island: assessing potential ecologic and climatic correlates. J Am Mosq Control Assoc. 2003; 19: 179–189 14524538

[39] Paz S, The West Nile virus outbreak in Israel (2000) from a new perspective: the regional impact of climate change. Int J Environ Health Res. 2006; 16: 1–13. 16507476 10.1080/09603120500392400

[40] Shaman J, Day JF, Stieglity M. Drought induced amplification and epidemic transmission of West Nile virus in southern Florida, J Med Entomol. 2005; 42: 134–141. 15799522 10.1093/jmedent/42.2.134

[41] Wang G, Minnis RB, Belant JL, Wax CL. Dry weather induces outbreaks of Human West Nile Virus infections. BMC Infect Dis. 2010; 10: 38 10.1186/1471-2334-10-38 20181272 PMC2841181

